# Long‐Term Trajectories of Multidimensional Outcomes in Psychosis Following Early Intervention During the Critical Period: The PEPP‐Montreal 10+ Study Protocol

**DOI:** 10.1111/eip.70258

**Published:** 2026-09-23

**Authors:** Olivier Percie du Sert, Joseph Ghanem, Vanessa McGrory, Karyne Anselmo, Kelly K. Anderson, Srividya Iyer, Ridha Joober, Jai Shah, Ashok Malla, Martin Lepage

**Affiliations:** ^1^ Douglas Research Centre Montreal Quebec Canada; ^2^ Department of Psychiatry McGill University Montreal Quebec Canada; ^3^ Department of Psychology McGill University Montreal Quebec Canada; ^4^ Department of Psychology Université du Québec à Montréal Montreal Quebec Canada; ^5^ Departments of Epidemiology & Biostatistics and Psychiatry Western University London Ontario Canada

**Keywords:** clinical protocols, healthcare outcomes, longitudinal studies, long‐term care, mental health recovery, psychotic disorders

## Abstract

**Introduction:**

While early intervention services (EIS) have demonstrated short‐term benefits, the long‐term maintenance of these gains remains uncertain. Individuals with first‐episode psychosis exhibit significant variability in their course of recovery. Understanding the risk and protective factors that shape long‐term outcome trajectories is essential to predicting and promoting sustained recovery. Here, we present the protocol for an extended 10‐year follow‐up study of social, mental, cognitive, and physical health outcomes, supplemented through linkage with health administrative databases to offer a holistic perspective on long‐term outcome trajectories.

**Aim:**

The primary objective of the study is to model the heterogeneity of long‐term trajectories across multiple outcome dimensions over a 10‐year follow‐up period using data‐driven methods.

**Method:**

The Prevention and Early Intervention Program for Psychoses (PEPP‐Montreal) is a well‐established, high‐fidelity EIS program operating within a universal healthcare system and an epidemiologically defined catchment area in South‐West Montréal, Canada. Between 2003 and 2018, PEPP‐Montreal conducted a detailed two‐year longitudinal assessment of 689 individuals aged 14–35 with first‐episode affective or non‐affective psychosis.

**Discussion:**

This study will help distinguish clinically meaningful subgroups, characterize their profiles, and identify early predictors of long‐term outcomes while providing insight into the mechanisms of change within trajectories. In particular, the study will assess whether trajectories shaped during the critical period are sustained over the long term. To our knowledge, this represents the most comprehensive investigation of long‐term trajectories following EIS in North America and is expected to lay the groundwork for optimizing EIS and developing personalized interventions.

## 1 Introduction

The first episode of psychosis (FEP) typically occurs during adolescence or early adulthood, derailing normal life trajectories (Crespo‐Facorro et al. 2021). Pioneering work from the pre‐antipsychotic era (E. Bleuler 1911, 1978; Ciompi 1988) established the heterogeneity of long‐term outcomes in schizophrenia, and studies conducted after the introduction of antipsychotics and deinstitutionalization confirmed this variability, with only a minority of patients achieving positive outcomes (Carpenter and Kirkpatrick 1988; Angst 1988; McGlashan 1988; Sartorius et al. 1996; Jobe and Harrow 2005; Peritogiannis et al. 2020).

The development of Early Intervention Services (EIS) in the late 1990s and early 2000s brought new hope for improving this prognosis (Malla et al. 2003; McGorry et al. 1996; Jørgensen et al. 2000; Johannessen et al. 2001; Craig et al. 2004; Kuipers et al. 2004; Chen 2004; Sim et al. 2004; Addington and Addington 2001). Their rationale rests on seminal research suggesting that the long‐term course of psychosis may be shaped during its earliest years (M. Bleuler 1978; Harrison et al. 2001), with studies linking longer duration of untreated psychosis to poorer outcomes proving particularly influential (Lally et al. 2017; Catalan et al. 2024; Salazar De Pablo et al. 2024; Solmi et al. 2023; Howes et al. 2021; Penttilä et al. 2014). Together, these findings gave rise to the critical period hypothesis (Birchwood et al. 1998), framing the 2–5 years surrounding onset as a window of opportunity during which timely, specialized and intensive care may produce lasting benefits (Birchwood et al. 1997). Accordingly, EIS were designed to reduce treatment delays and deliver comprehensive, integrated, evidence‐based pharmacological and psychosocial interventions through multidisciplinary teams and assertive case management (McGorry et al. 1996; Addington et al. 2013). At the end of treatment, EIS consistently outperform standard care across clinical, social, and economic outcomes (Bird et al. 2010; Harvey et al. 2007; Marshall and Rathbone 2011; Álvarez‐Jiménez et al. 2011; Puntis, Minichino, De Crescenzo, Cipriani, et al. 2020; Correll et al. 2018). Although benefits were hoped to persist beyond the period of specialized care, preliminary evidence from clinical trials suggests they largely dissipate within 3 years of discharge, with the possible exception of hospitalization days (Puntis, Minichino, De Crescenzo, Harrison, et al. 2020; Salazar De Pablo et al. 2026). This apparent fading of benefits raises the question of how to preserve the hard‐won gains achieved through EIS over the longer term?

Yet the research needed to answer this question remains limited and its findings mixed. Most long‐term studies predate EIS (Volavka and Vevera 2018; Peralta et al. 2022; O'Keeffe et al. 2019; Tramazzo et al. 2024), and only a handful have followed FEP patients beyond 5 years from entry into prototypical, high‐fidelity programs (Chan et al. 2019). Mihalopoulos et al. reported lower positive symptoms at the 8‐year follow‐up of the Early Psychosis Prevention and Intervention Centre (EPPIC) (Mihalopoulos et al. 2009), while the 15‐year follow‐up is ongoing (Cotton et al. 2021). In contrast, Chan et al. found no difference in positive or negative symptoms, nor in rates of clinical and functional remission at the 10‐year follow‐up of the Early Assessment Service for Young People with Psychosis (EASY). However, they did report lower depressive symptoms and suicidal behaviours along with fewer and shorter hospitalizations and longer periods of employment (Chan et al. 2015). Hegelstad et al. similarly found no differences in clinical remission but reported higher employment rates at 10 years of the Early Treatment and Intervention in Psychosis (TIPS) (Hegelstad et al. 2012). Finally, the long‐term follow‐ups of the OPUS trial revealed no significant differences between EIS and treatment as usual in functioning, positive and negative symptoms, or clinical remission and recovery at 10 (Secher et al. 2015), and 20 years (Hansen et al. 2023), though EIS patients spent fewer days in hospital and supported housing.

Beyond scarcity, long‐term follow‐up studies face specific methodological challenges that limit the interpretability, reproducibility, and generalizability of their findings, including high attrition, small sample size, poor representativeness of retained cohorts, numerous confounders, and insufficient statistical power (Percie du Sert et al. 2026). These limitations may partly account for the limited success of meta‐analyses in identifying malleable predictors of long‐term clinical and functional recovery (Correll et al. 2018; Hansen et al. 2023; Molstrom et al. 2022; Catalan et al. 2021; Jaaskelainen et al. 2013; van Dee et al. 2023). Despite insight into some prognostic factors, none has proven sensitive or specific enough to estimate, let alone improve, an individual's chance of recovery (Dazzan et al. 2020), leaving clinicians with limited guidance for treatment planning and prognosis. These shortcomings may also reflect the considerable heterogeneity of outcomes (Shah et al. 2022) among individuals with FEP whose responses to EIS differ markedly (Percie du Sert et al. 2025). One size does not fit all, and personalized treatment strategies cannot be developed without first accounting for this heterogeneity. Research relying solely on group average lacks the resolution to identify clinically meaningful subgroups, inform targeted interventions, or yield accurate prognoses.

Patient heterogeneity has long prompted the exploration of subgroups defined by clinical or biological features. Early efforts to dissect heterogeneity included the delineation of Type I and Type II schizophrenia (Crow 1985) as well as deficit and non‐deficit schizophrenia (Carpenter Jr 1994) based on the prominence of positive and negative symptoms. Later, the clinical staging model sought to identify more clinically homogeneous subgroups along a neurodevelopmental continuum, ranging from clinical high risk to chronic and enduring schizophrenia (McGorry et al. 2007). More recently, the Research Domain Criteria (RDoC) (Insel et al. 2010; Insel 2014) and the Hierarchical Taxonomy Of Psychopathology (HiTOP) (Kotov et al. 2017, 2020, 2021) have proposed dimensional frameworks designed to address the limitations of traditional categorical diagnostic systems, including within‐disorder heterogeneity, with a focus on neurobiological, neurocognitive and psychopathological dimensions. Heterogeneity can be effectively addressed using data‐driven approaches like longitudinal latent growth modelling (LGM). LGM captures both within‐ and between‐individual variability, allowing it to model outcome trajectories with distinct patterns of change over time, and distinguish underlying differences between homogeneous subgroups of patients (Van Der Nest et al. 2020). Habtewold et al. reviewed data‐driven studies in schizophrenia spectrum disorders and identified very few conducted in FEP cohorts receiving EIS, with fewer still extending to follow‐up beyond 5 years (Habtewold et al. 2020). Only two studies were available, both reporting trajectories of positive and negative symptoms indicating that up to 50% of patients may preserve the gains initially achieved through EIS (Chan et al. 2020; Starzer et al. 2023). Although preliminary, this points to a potential for consolidating EIS benefits over time, and underscores the need for long‐term follow‐up research centred on longitudinal analyses of individual trajectories, capable of identifying who maintains these gains, who does not, and why.

We present the protocol for an extended 10‐year follow‐up study of the PEPP‐Montreal cohort, comprising a large sample of individuals with FEP who previously received years of EIS. The primary objective of the study is to model the heterogeneity of long‐term trajectories across multiple outcome domains over a 10‐year follow‐up period using data‐driven methods. Specifically, the study aims to (1) Identify latent trajectories of functional, clinical, cognitive and healthcare outcomes; (2) Characterize the social, mental, cognitive, physical health, and healthcare profiles of individuals associated with each trajectory (e.g., disability, severity, comorbidity, and mortality), and further describe each trajectory in terms of its course (e.g., stability, progression, deterioration, or transition over time); and (3) Assess the prognostic value of these trajectories by identifying early risk and protective factors associated with trajectory membership, rates of change and long‐term outcomes.

## 2 Methods

### 2.1 Design

The Prevention and Early Intervention Program for Psychosis (PEPP) is a well‐established, high‐fidelity EIS at the Douglas Mental Health University Institute in Montreal, Canada. Launched in 2003 as a publicly funded catchment area‐based service covering a population of 350 000, PEPP‐Montréal has served over 1100 patients to date. A detailed description of the clinical program, along with the 2‐ and 5‐year outcomes, is reported elsewhere (Malla et al. 2003, 2006, 2017, 2014; Iyer et al. 2015). This service was established as a clinical‐research program and implemented a comprehensive assessment protocol between 2003 and 2018. During this period, 689 individuals with FEP were systematically characterized over the course of the two‐year EIS follow‐up. Assessments were conducted at baseline and at 1, 2, 3, 6, 9, 12, 18, 24 months and 5 years. Fidelity to the EIS delivery model was established through monitoring of case manager‐to‐patient ratios, minimum number of clinical contacts per month and the availability of a range of psychosocial interventions (Nolin et al. 2016; Bertulies‐Esposito et al. 2022). Participants are now being invited to take part in an extended naturalistic observational study with a 10‐year follow‐up assessment. The secondary use of data, access to medical records and contact information, along with participant recontact without explicit prior consent received ethical approval in accordance with the Tri‐Council Policy Statement of Ethical Conduct (articles 5.5A; 5.6) (Canadian Institutes of Health Research NSaERCoC, and Social Sciences and Humanities Research Council of Canada 2022).

### 2.2 Recruitment

#### 2.2.1 Inclusion and Exclusion Criteria

At the time of admission to PEPP‐Montreal, patients met the following inclusion criteria: aged between 14 and 35 years; DSM‐IV diagnosis of psychotic disorder, including both schizophrenia‐spectrum or affective psychoses; no or minimal prior antipsychotic treatment (< 30 days). Excluded patients were those with organic or drug‐induced psychosis, a history of a central nervous system disease, an IQ below 70, and inability to provide informed consent or speak either English or French fluently. All former patients are eligible to participation in the current study, regardless of remission status or concurrent substance use disorder.

#### 2.2.2 Tracing and Contact Strategy

**FIGURE 1:**
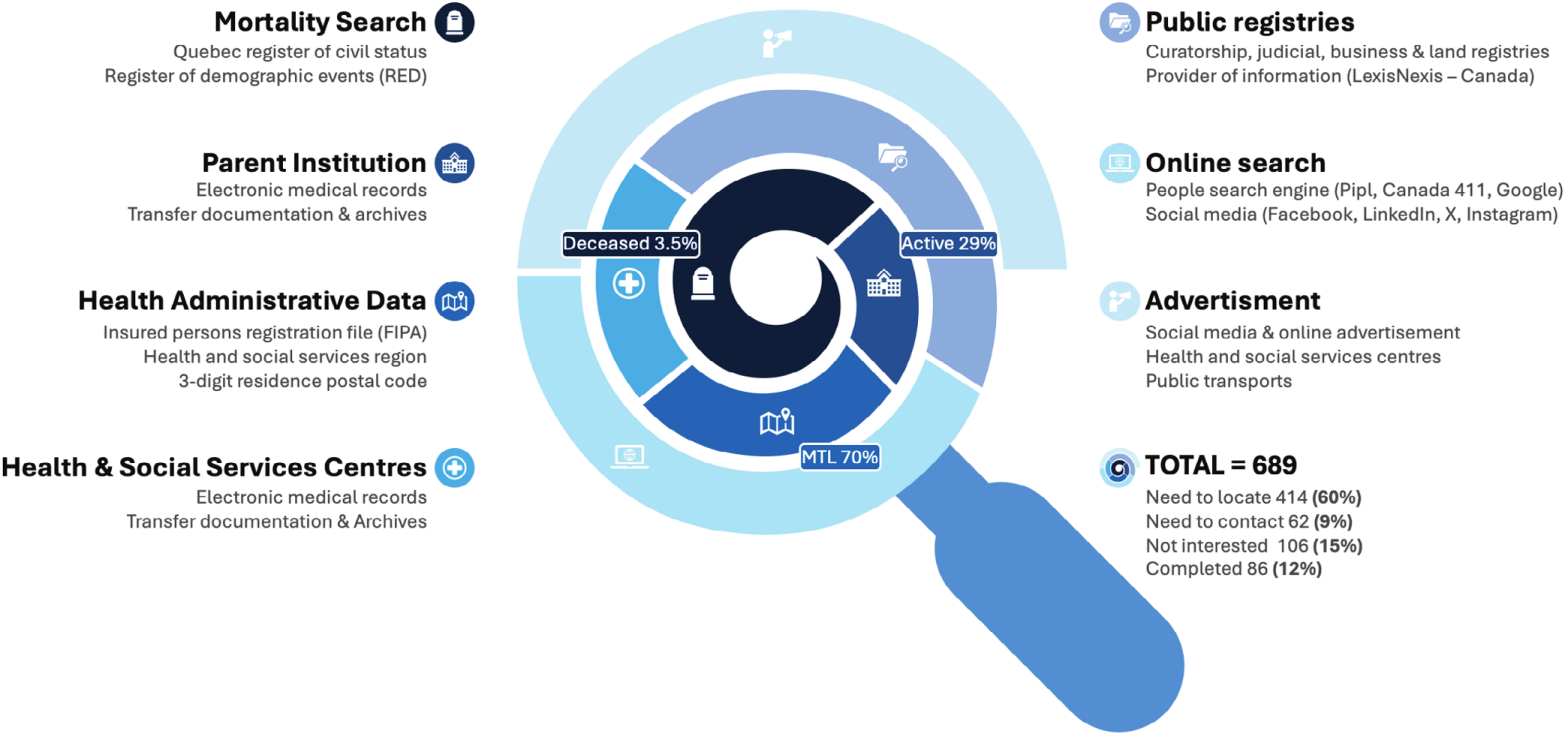
Tracing strategies for locating and reconnecting with participants.

As the only EIS within the catchment area, PEPP‐Montreal has the potential to provide a representative sample. Recruiting a representative sample of patients who have received EIS is the most important and challenging aspect of the current protocol. Participant tracing and contact retrieval will be carried out using a stepwise strategy aligned with best practices (Ou et al. 2020; Dimakos et al. 2023) and summarized in Figure 1. First, deceased individuals will be identified through consultation of public death records. For remaining participants, residential addresses, phone numbers, and email addresses will be retrieved by searching electronic medical records, archives, and transfer documentation from the parent institute. Initial contact attempts will be made using the last known contact information, beginning with individuals who are still receiving services at the parent institute. Next, provincial health administrative databases will be consulted to determine the most recent region of residence (3‐digit postal codes) and the corresponding health and social services region. Access to electronic medical records systems and archives will then be requested from the relevant health and social services centres to update contact information when available. For remaining participants, public registries, such as curatorship, judicial, business and land ownership records may be consulted with the support of authorized information providers (e.g., LexisNexis) that aggregate such data, in an effort to locate individuals. Contact information will also be retrieved through online searches of social media accounts (e.g., LinkedIn, Facebook, X, Instagram) and other online profiles, using people‐search engines such as Pipl, Canada411 and Google. To verify and confirm digital identity, information will be corroborated using participants' first and last names, date of birth, and previous addresses. When all existing contact methods will have been exhausted, individuals will be contacted through private messaging systems on their most recently updated social media platform as a last resort, following established recommendations (Dimakos et al. 2023; Andrawes et al. 2024). Finally, targeted advertisements will be used to encourage self‐referral, including posters and paid ads, both online and in the community, on platforms such as Google and Facebook, as well as in public transit spaces and health and social service settings. Recruitment is currently ongoing, with 88 participants already enrolled and a mean follow‐up of 13.7 years [range: 6.4–22.4] from admission. Preliminary investigations revealed that over 70% of the sample reside within the health and social services region of Montreal, while approximately 30% still receive care directly at the parent institute. Recruitment is expected to be completed by early 2027.

Consecutive patients previously admitted to PEPP‐Montreal are being sent a generic invitation letter that does not disclose any confidential information related to mental health or specific services. The letter includes a QR code linking to a form where prospective participants can indicate their interest in the study and update their contact information. Letters are sent to the last known address, or an updated address if available. When possible, research assistants follow up with a phone call, making up to five attempts within 1 month at varying times of day. If necessary, further attempts are made at 3, 6 months and 1 year before the participant is considered lost to follow‐up. Due to ethical constraints, home visits and tracing through secondary contacts (e.g., friends, neighbours or employers) are not pursued. To maximize engagement and retention, participants are offered financial incentives, flexible scheduling (e.g., outside of office hours), and multiple data collection options (i.e., in‐person or remote), transport accommodations, with phone call reminders, in line with published recommendations (Deckler et al. 2022; Teague et al. 2018; Ewing et al. 2018).

### 2.3 Assessment

**FIGURE 2:**
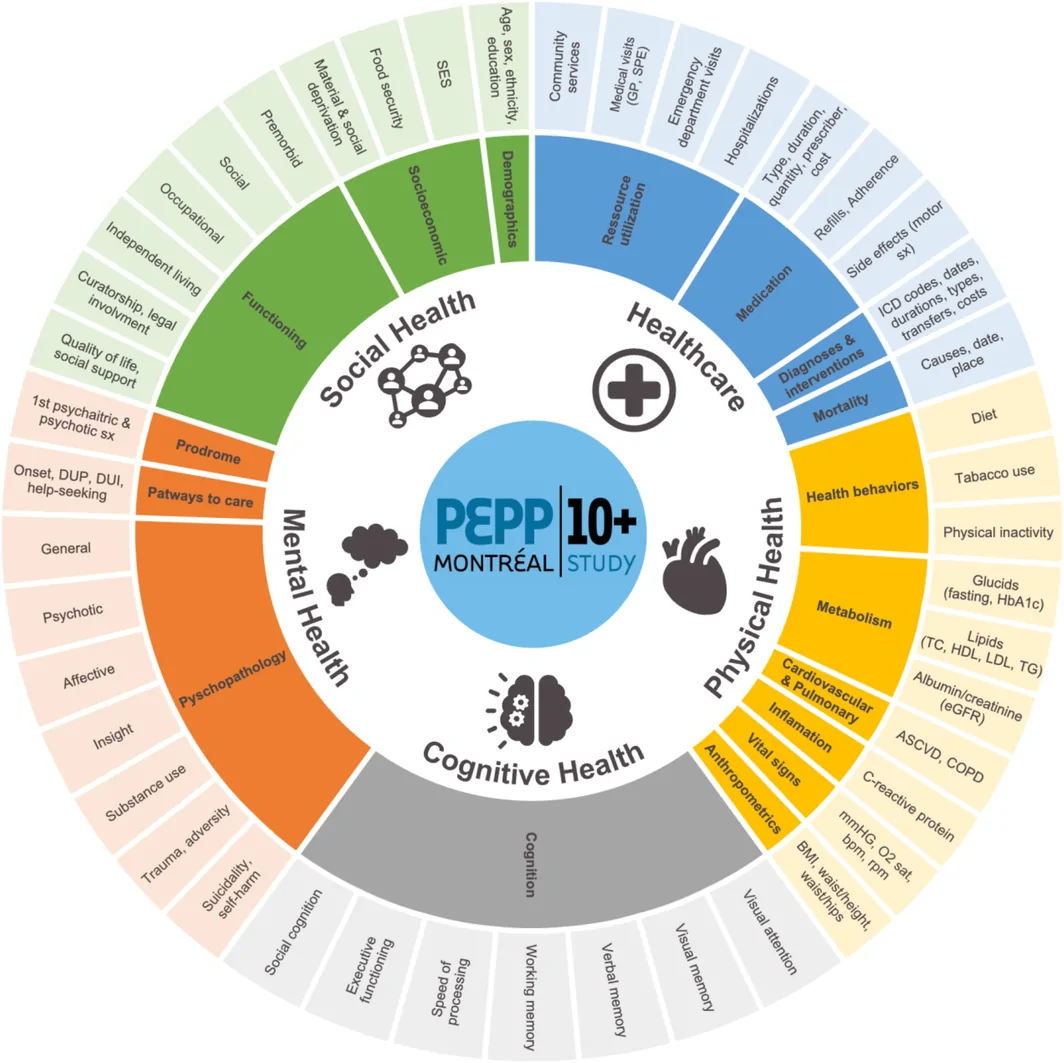
Overview of assessment protocol. ASCVD, Atherosclerotic Cardiovascular Disease; bpm, Beats per Minute; DUI, Duration of Untreated Illness; DUP, Duration of Untreated Psychosis; eGFR, Estimated Glomerular Filtration Rate; GP, General Practitioner; HDL, High‐Density Lipoprotein; LDL, Low‐Density Lipoprotein; rpm, Respirations per Minute; SES, Socioeconomic Status; SPE, Specialized Psychiatric Evaluation; sx, Symptoms; TC, Total Cholesterol; TG, Triglycerides.

Given that the etiology of psychotic disorders involves a range of sociodemographic, clinical, cognitive, biological, neurobiological and environmental factors, a multidimensional approach is expected to provide a better characterization of trajectories and greater predictive accuracy than using measures from a single data modality (Coutts et al. 2023; Kas et al. 2025). The measures and domains included in the protocol were informed by findings from the Meaningful Assessment Protocol (MAP), a scoping review that identified key domains, measures, and metrics in EIS research to support the harmonization of assessment practices (Ferrari et al. 2023) and facilitate comparisons. In line with the principles of measurement‐based care and practice‐based research, the current protocol balances replication of the original battery, to ensure consistency and comparability over time, with the integration of newer scales and methods. Similarly to the U.S. Early Psychosis Intervention Network's Core Assessment Battery (CAB‐EPINET) (George et al. 2020), a harmonized protocol developed through expert consensus to assess key domains relevant to psychosis at the patient level, the current protocol includes assessments of social, mental, cognitive, physical health, healthcare, and recovery. It includes clinician‐rated and patient‐reported outcome measures, as well as performance‐based, biological measures and administrative data. The full assessment will be administered over three separate two‐hour visits. The assessed domains are summarized in Figure 2. A full description of the assessment protocol, including all scales and interviews administered at each time point, is provided in Table 1.

**TABLE 1:** Scales and assessment measures.

	Baseline *n* = 689	Months 1, 2, 3	Months 6, 9	Months 12	Months 18, 24	Years 5 *n* = 220	Years 10
**Social health**							
*Sociodemographics*							
Age, sex, ethnicity, education (SD)	SD					SD	SD
*Socioeconomic*							
Hollingshead 4‐Factor Index (SES)	SES					SES	SES
Household Food Security Survey Module (HFSSM)							HFSSM
Material/Social deprivation index (MSDI)	MSDI			MSDI	MSDI		MSDI
*Functioning*							
Premorbid Adjustment Scale (PAS)		PAS					
Strauss‐Carpenter (SC)	SC			SC	SC	SC	SC
Social/Occupational Functioning Assessment Scale (SOFAS)	SOFAS			SOFAS	SOFAS	SOFAS	SOFAS
Multidimensional Scale of Perceived Social Support (MSPSS)	MSPSS			MSPSS	MSPSS		MSPSS
WHO Life Chart Schedule (LCS)							LCS
**Mental health**							
*Prodrome and Pathways to care*							
Course of Onset and Relapse Schedule (CORS)		CORS					
*Psychopathology*							
Structured Clinical Interview for DSM‐IV (SCID‐IV)		SCID		SCID			SCID
Positive and Negative Syndrome Scale (PANSS)	PANSS	PANSS	PANSS	PANSS	PANSS	PANSS	PANSS
Scale for Assessment of Positive Symptoms (SAPS)	SAPS	SAPS	SAPS	SAPS	SAPS	SAPS	SAPS
Scale for Assessment of Negative Symptoms (SANS)	SANS	SANS	SANS	SANS	SANS	SANS	SANS
Hamilton Anxiety Scale (HAS)	HAS	HAS	HAS	HAS	HAS		HAS
Calgary Depression Scale (CDS)	CDS	CDS	CDS	CDS	CDS		CDS
Young Mania Rating Scale (YMRS)	YMRS	YMRS	YMRS	YMRS	YMRS		YMRS
Scale to Assess Unawareness of Mental Disorder (SUMD)	SUMD	SUMD	SUMD	SUMD	SUMD		SUMD
The Chemical Use, Abuse, and Dependence Scale (CUAD)	CUAD	CUAD	CUAD	CUAD	CUAD	CUAD	CUAD
Childhood Trauma questionnaire (CTQ)							CTQ
PTSD Checklist for DSM5 (PCL‐5)							PCL‐5
Life Events Checklist for DSM‐5 (LEC‐5)							LEC‐5
Columbian Suicide Severity Rating Scale (CSSRS)							CSSRS
**Cognitive health**							
*Cognition*							
Wechsler Abbreviated Scale of Intelligence (WASI)		WASI‐II					
CogState Research Battery (CSRB)		CSRB		CSRB			CSRB
**Physical health**							
*Physical examination*							
Vital signs—BP, HR, RR, O2sat (VS)							VS
Anthropometrics—height, weight, waist ratios (AMtrics)	AMtrics	AMtrics	AMtrics	AMtrics	AMtrics		AMtrics
Extrapyramidal Symptoms Rating Scale (ESRS)	ESRS	ESRS	ESRS	ESRS	ESRS		ESRS
*Blood test*							
Lipids profile—TC, LDL, HDL, TGs (Lipids)							Lipids
Glucose profile—fasting, HbA1c (Glucids)							Glucids
Kidney profile—creatinine/albumin (eGFR)							eGFR
Inflamation profile—C‐reactive Protein (CRP)							CRP
*Cardiovascular and Pulmonary*							
Compressive‐Obstructive Pulmonary Disorders (COPD)							COPD
Atherosclerotic Cardiovascular Disease Risk (ASCVD)							ASCVD
*Health Behaviours*							
Short Form Health Survey (SF‐12)							SF‐12
Heaviness of smoking Index (HSI)							HIS
Mediterranean Diet Adherence Screener (MEDAS)							MEDAS
Simple Physical Activity Index Questionnaire (SIMPAQ)							SIMPAQ
**Healthcare (Administrative data)**							
*Quebec Health Insurance Board (RAMQ)*							
Insured Persons Information (FIPA)			FIPA				
Medical services (MOD)			MOD				
Pharmaceutical services (MED)			MED				
*Ministry of Health and Social Services (MSSS)*							
Register of Demographic Events (RED)			RED				
Clientele and Services Information System for CSSSs (I‐CLSC)			I‐CLSC				
Emergency Database (BDCU)			BDCU				
Data for the Study of Hospital Clientele (MED‐ECHO)			MED‐ECHO				
Hospital Performance (APR‐DRG)			APR‐DRG				
**Endpoints**							
Treatment Response (TRRIP)	TRRIP						
Treatment Resistance—early and late (TRRIP)	Early—TR				Late—TR		Late—TR
Remission—clinical and functional (RSWG)					RSWG		RSWG
Relapse (REL)						REL	REL
Recovery—clinical and functional (RECOV)							RECOV
Wisconsin Quality of Life (WQoL)							WQoL
Personal Recovery Assessment Scale (RAS)							RAS

#### 2.3.1 Social Health

*Sociodemographics*: information was collected at admission and updated at the 10‐year follow‐up. Socioeconomic status is assessed using the Hollingshead Four‐Factor Index (SES) (Hollingshead 1975), food security is measured with the Household Food Security Survey (HFSS) (Gulliford et al. 2006), and area‐level deprivation is estimated using the Material and Social Deprivation Index (Institut National de Santé Publique du Québec 2021). *Functioning*: Premorbid academic and social functioning from childhood through early adolescence is assessed retrospectively with the Premorbid Adjustment Scale (PAS) (Cannon‐Spoor et al. 1982). The Social and Occupational Functioning Assessment Scale (SOFAS) (Morosini et al. 2000) is used to assess global functioning, independent of the severity of psychiatric symptoms. Records of curatorship along with the nature of the responsibilities will be obtained from the Quebec Public Register of Representation Measures. Occupational, role, and housing history as well as social contacts at follow‐up are self‐reported using the WHO Life Chart Schedule (LCS) (Linszen et al. 1998) and Strauss‐Carpenter Outcome Scale (SCOS) (Strauss and Carpenter 1972). Perceived social support from family, friends, and significant others is measured with the Multidimensional Scale of Perceived Social Support (MSPSS) (Zimet et al. 1988).

#### 2.3.2 Mental Health

*Prodrome, Pathways to Care and Diagnosis*: Information including prodromal symptoms and the duration of untreated psychosis and illness were collected using the Circumstances of Onset and Relapse Schedule (CORS) and validated operational criteria (Malla et al. 2006). Diagnosis was initially established at admission using the SCID‐IV (First 1997) and confirmed at the 1‐ and 10‐year follow‐ups. *Psychopathology*: Assessments are repeated at 10‐year follow‐up including the severity of positive and negative symptoms assessed using the Scale for Assessment of Positive (SAPS) (Andreasen 1984) and Negative Symptoms (SANS) (Andreasen 1984). Anxiety, depression, and mania are evaluated using the Hamilton Anxiety Scale (HAS) (Riskind et al. 1987), the Calgary Depression Scale (CDS) (Addington et al. 1993), and the Young Mania Rating Scale (YMRS) (Young et al. 1978), respectively. In addition, childhood trauma and post‐traumatic stress symptoms are assessed using the Childhood Trauma Questionnaire (CTQ), the PTSD Checklist for DSM‐5 (PCL‐5), and the Life‐event checklist (LEC‐5). Suicidal behaviours and history of previous suicide attempts are recorded with the Columbia suicide severity rating scale (C‐SSRS) (Posner et al. 2011). Insight is rated with the Scale to Assess Unawareness of Mental Disorder (SUMD) (Amador et al. 1993), and substance use is documented using the Chemical Use, Abuse, and Dependence Scale (CUAD) (McGovern and Morrison 1992). *Medication*: Antipsychotic use was recorded throughout treatment at PEPP and is collected at the 10‐year follow‐up through medical records review, with dosages converted to chlorpromazine equivalents based on established guidelines (Leucht et al. 2020), while medication adherence is assessed through self‐report using standard thresholds (Velligan et al. 2005). Motor side effects, including tardive dyskinesia, dystonia, parkinsonism, and akathisia, are evaluated using the Extrapyramidal Symptom Rating Scale (ESRS) (Chouinard and Margolese 2005).

#### 2.3.3 Cognitive Health

Cognitive performance was assessed at baseline and 1 year across all seven MATRICS domains (i.e., verbal memory, visual memory, working memory, visual attention, processing speed, executive functioning, and social cognition) (Nuechterlein et al. 2008) and is re‐evaluated at the 10‐year follow‐up using the computerized CogState Research Battery (Pietrzak et al. 2009).

#### 2.3.4 Physical Health

While we aim to provide a comprehensive overview of body systems and functions, we focus specifically on screening for metabolic syndrome, given its high prevalence in psychosis, the presence of multiple contributing risk factors, and its strong association with comorbid metabolic, cardiovascular, and pulmonary disorders (Rodrigues et al. 2021). *Physical examination* captures vital signs (blood pressure, heart and respiratory rates, oxygen saturation) and anthropometric measures (body mass index, waist‐to‐hip ratio, and waist‐to‐height ratio) to assess overweight/obesity and estimate body composition. *Laboratory analyses* include glycated haemoglobin (HbA1c) and fasting blood glucose to screen for diabetes, a standard lipid panel (total cholesterol, HDL, LDL, and triglycerides) for dyslipidemia, albumin‐to‐creatinine ratio to estimate the glomerular filtration rate, and C‐reactive protein as an inflammatory marker. Cardiovascular risk is estimated using Framingham risk scores as per the Canadian Cardiovascular Society recommendations (Grundy et al. 2004; Pearson et al. 2021) while the risk for Chronic Obstructive Pulmonary Disease (COPD) is estimated using the self‐administered COPD population screener (Martinez et al. 2008). *Behavioural and environmental risk factors* are assessed, including tobacco use with the Heaviness of Smoking Index (HSI) (John et al. 2004), physical inactivity with the Simple Physical Activity Questionnaire (SIMPAQ) (Wareham et al. 2003), diet quality with the Mediterranean Diet Adherence Screener (MEDAS) (Papadaki et al. 2018). Perceived physical health is measured using the single‐item general self‐rated health (GSRH) measure (Jurges et al. 2008) and the Medical Outcomes Study Short Form‐12 (SF‐12).

#### 2.3.5 Healthcare and Administrative Data

Given the expected loss to follow‐up and the substantial gap between the 2‐, 5‐ and 10‐year assessments, health administrative data are used to supplement primary data collection and to provide continuous information for the entire cohort over the full follow‐up period. This approach also reduces participant burden while providing reliable, real‐world data that would otherwise be difficult to obtain via self‐report. The data, collected and maintained by the Quebec Ministry of Health and Social Services (MSSS) and the Provincial Health Insurance Board (RAMQ), are accessed through the Quebec Institute of Statistics (ISQ), and have been shown to have good quality and reliability for research purposes (Tamblyn et al. 1995). Linkage with the PEPP cohort was performed using health insurance number, full name, parental names, sex, and date of birth, resulting in 95% of individuals successfully matched. Data were extracted from the date of entry at PEPP‐Montreal until the date of 10‐year follow‐up (or the most recent date of data availability at the time of extraction).

Health administrative data include detailed information on emergency department visits, hospitalizations, visits to specialists and general practitioners, interventions from local community service centres, medication prescriptions (for those covered under the public drug plan), and mortality data, including date and causes of death. For each service, the databases include detailed information on the service providers, service dates, associated diagnoses, interventions performed, and related costs. Mental Health Care Resource Utilization (MHCRU) will be operationalized as the total number of outpatient psychiatric‐related contacts with general practitioners, psychiatrists, and emergency departments, as well as the number and duration of psychiatric hospitalizations. These will be identified based on the provision of mental health services using ICD codes, in accordance with the CIHI Mental Health and Substance Use Diagnosis Code Groupings (Canadian Institute for Health Information 2024). MHCRU will be computed as count variables for each follow‐up year and as cumulative totals, beginning from the index date of admission to PEPP‐Montreal services and ending at the date of data extraction or right‐censored at 10 years.

In addition, data were requested for three control groups: (1) Non‐EIS controls: individuals who experienced FEP but never received EIS, (2) Mental health controls: individuals with any non‐psychotic psychiatric disorder, and (3) Physical health controls: individuals with any physical health condition and no lifetime history of psychiatric diagnosis. Case identification was conducted using a validated algorithm (Anderson et al. 2019; Kurdyak et al. 2015), individuals were then randomly selected by ISQ statisticians and matched to the PEPP cohort based on sociodemographic characteristics, service catchment area, and time period of service use. To further address potential disparities in clinical characteristics and other confounding variables across groups, we will apply propensity score weighting to improve comparability with controls (Kurz et al. 2025). Health administrative data will be key in determining the representativeness of our recruited sample and essential for characterizing care trajectories using the multidimensional ‘6 W’ model (Vanasse et al. 2018), which considers patient attributes and health conditions (‘who’ and ‘why’), types of healthcare providers (‘which’), care settings (‘where’), treatments received (‘what’), and timing of services (‘when’). Selection criteria for the control groups, including diagnostic codes, are detailed in Table S1.

#### 2.3.6 Turning Points and Endpoints: Response, Resistance, Remission and Recovery

*3‐month*: Early treatment response will be defined as a ≥ 50% reduction in the severity of positive and/or negative symptoms within the first three months of follow‐up at PEPP‐Montreal (Zhu et al. 2017). *2‐year*: Early and late treatment resistance will be operationalized according to criteria established by the Treatment Response and Resistance in Psychosis (TRRIP) Working Group (Howes et al. 2016). Clinical remission will be defined using the Remission in Schizophrenia Working Group (RSWG) criteria, as minimal symptom severity (i.e., scores ≤ 2 on all global items of the SAPS and SANS, excluding the attention subscale) sustained for at least 6 months (Andreasen et al. 2005). Functional remission will be defined as achieving a SOFAS score > 60 (Harrison et al. 2001; Morosini et al. 2000). *5‐year*: Relapse will be defined as any emergency department visit or hospitalization for any psychiatric reason in individuals who had previously achieved clinical remission (Cristarella et al. 2022). *10‐year*: Clinical recovery will require meeting criteria for clinical remission along with the absence of psychiatric hospitalizations or emergency department visits for mental health reasons over a continuous 2‐year period. Functional recovery will be defined as meeting criteria for functional remission, along with having meaningful social contact (Strauss‐Carpenter Outcome Scale, item 2 score > 1), living independently, and being engaged in full‐time employment, education, training, or homemaking for a minimum of 2 years, or being retired. Individuals will be considered in full recovery if they met criteria for both clinical and functional recovery. Quality of life, including satisfaction with housing, occupation, relationships, and health, is evaluated using the Wisconsin Quality of Life Index (WQOL). Personal recovery, as an ongoing process, is measured using the Recovery Assessment Scale (RAS), which captures key dimensions such as hope, self‐determination, and goal orientation (Corrigan et al. 2004).

## 3 Analyses

Descriptive statistics, including point estimates and confidence intervals will be calculated to summarize the data on all participants. Primary analyses will focus on modelling and characterizing trajectories of functioning (SOFAS), while secondary analyses will investigate trajectories of clinical symptoms, cognitive performance, and patterns of service use as well as their interrelationships using similar methodology. All analyses will be performed using R (R Core Team 2026) and Mplus (Muthén and Muthén 2017) software. The MplusLGM (Percie du Sert and Unrau 2025) and MplusAutomation packages for R (Hallquist and Wiley 2018) will be used to automate the iterative model‐fitting procedure and the manual 3‐step approach for analysis of covariates. Statistical significance will be set at *p* < 0.05. Missing data will be handled using full information maximum likelihood (FIML) estimation when appropriate, along with sensitivity analyses to evaluate the impact of non‐random missing data.

### 3.1 Trajectory Analyses

Longitudinal latent growth modelling (LGM) will be used to examine long‐term trajectories of outcomes (Nagin 2005). Trajectory modelling will be conducted following the procedure outlined by Van Der Nest et al. (2020) and the Framework to construct and interpret latent class trajectory modelling (Lennon et al. 2018). This involves fitting increasingly less constrained LGMs in a systematic fashion to determine the optimal and most parsimonious set of model parameters. First, a single‐class growth curve model (GCM) will be estimated to represent the sample mean trajectory of outcome over time, against which subsequent models will be compared. Second, class enumeration will be performed by fitting a series of group‐based trajectory models (GBTM) with an increasing number of classes and fixed residual variance. The maximum number of latent classes to be tested will be guided by prior research findings (Habtewold et al. 2020). Third, the model with the optimal class structure will be extended in a latent class growth analysis (LCGA) by allowing free estimation of the residual variance across time, classes, and both. Fourth, class‐invariant and class‐variant random effects will be added stepwise in growth mixture models (GMM) by allowing the variance and covariance of the growth factors to be estimated and to vary across classes. To mitigate the risk of local maxima, each model will be rerun with twice the number of random starts (starting with a set of 500 and 500/4) until the best log‐likelihood value is replicated within and between 2 runs.

In addition, piecewise LGMs will be conducted to examine whether rates of change differ across distinct time intervals. This approach will allow for the estimation of separate growth factors for each time period, testing potential turning points at 3 months, 1, 2, and 5 years. Informed by previous univariate models, multivariate LGMs will be explored, including joint‐trajectory modelling with parallel and sequential processes to investigate interrelationships between independent trajectories of functional and clinical symptoms, cognitive performance, and service use, as well as 2‐, 5‐, and 10‐year trajectories. Conditional probability tables will be generated to estimate the likelihood of an individual following a given trajectory *i*, conditional on their membership in a different trajectory *j*. To provide a comprehensive view of the overall long‐term course of psychosis, a multidimensional LGM will also be estimated integrating multiple outcome domains into a single set of trajectories. Finally, to account for variation in assessment timing due to rolling admissions, differing follow‐up windows, and gaps in the timeline, sensitivity analyses will be conducted using individually varying times of observation and freely estimated timescores. This approach improves model accuracy by reflecting the actual timing of measurements, reduces bias from assuming uniform intervals, and enhances the robustness of longitudinal inferences.

Model selection will be determined according to the Bayesian information criterion (BIC), the scaled entropy (sE), the average posterior probabilities (APPA), and the bootstrap likelihood ratio test (BLRT). A significant BLRT indicates that a K class model has a significantly better fit than a K‐1 class model. Lower BIC values suggest a more parsimonious model, higher entropy (> 0.5) values imply greater classification certainty of individuals, while higher APPA (> 0.7) are indicative of a good fit. Class separation will be estimated using degrees of separation (DoS > 0.5) and overlap coefficients (OVL < 0.8) (Van Der Nest et al. 2025). Aside from fit statistics, interpretability will also be taken into consideration, and models with classes accounting for less than 5% of the sample will be rejected. Given the number of available timepoints, a cubic trend will be assumed, and the polynomial order for the best‐fitting model will be refined by iteratively removing the highest non‐significant term based on Wald tests. While heterogeneity in the overall distribution of the data is expected, normality will only be assessed within classes of the final model using the multivariate skewness and kurtosis test (B. Muthén 2003). If required, corrected distributions will be used accordingly to accommodate excessive skewness or kurtosis such as provided by Mplus (Muthén and Asparouhov 2015). Models will be reported in accordance with the guidelines for reporting on latent trajectory studies checklist to ensure transparency, reproducibility, and methodological rigour (Van De Schoot et al. 2017).

### 3.2 Analyses of Covariates

The association of trajectory membership with baseline predictors, time‐varying covariates and distal outcomes will be investigated following a manual 3‐step approach (Vermunt 2010; Asparouhov and Muthén 2014). First, an unconditional model with no covariates will be estimated including class enumeration, model selection and refining of polynomial order. Second, misclassification rates converted in logit values will be extracted from the best‐fitting model. Third, covariates will be included in the model while manually fixing the misclassification rates, ensuring that class membership remains stable and is not influenced by the addition of new model parameters (Wickrama et al. 2021). Covariates will be identified based on their relevance in the literature, particularly those associated with functional and clinical remission and recovery (Molstrom et al. 2022; van Dee et al. 2023; Habtewold et al. 2020; Santesteban‐Echarri et al. 2017). Multinomial logistic regressions will be used to identify predictors of latent class membership. Odds ratios will be reported for the “better” trajectories in reference to the “worse” trajectory, as the exponentiation of the logistic coefficients. To further examine dynamic associations, mixture regression between the growth factors of parallel and sequential processes will be used to evaluate the effects of time‐varying covariates on the rates of change within trajectories. Latent Transition Analysis (LTA) will be employed to estimate the probability of transitioning between classes over time, while identifying the specific timepoints at which covariates have the greatest influence on these transitions. For distal outcomes, Wald chi‐square tests will assess differences in means and thresholds across latent trajectoriy endpoints. Standardized mean differences will be calculated as the ratio of estimated group mean differences to the within‐sample standard deviation. Bonferroni corrections will be applied to control for multiple comparisons. Finally, a multivariable prognostic model will be constructed using an augmented backward elimination procedure, guided by both statistical significance and change‐in‐estimate criteria (Dunkler et al. 2014), in accordance with the updated guidance for reporting clinical prediction models that use regression or machine learning methods (TRIPOD + AI) (Collins et al. 2024).

### 3.3 Sensitivity Analyses

To assess sample representativeness, we will compare participants who were successfully enrolled with those who refused participation or were lost to follow‐up on a range of observed variables, including sociodemographic characteristics, baseline clinical measures, and MHCRU, leveraging control groups and administrative data linkage at the 10‐year follow‐up. If evidence suggests that data are not missing at random, we will explore alternative modelling approaches, including selection models (e.g., Diggle–Kenward) or pattern‐mixture models (Muthén and Muthén 2017) incorporating auxiliary variables identified in preliminary analyses. These analyses will help contextualize potential selection and attrition bias beyond what can be addressed using FIML under missing‐at‐random assumptions. To further evaluate the robustness of our findings to missing data, we will also replicate trajectory analyses excluding participants with more than 30% missing data across follow‐up.

### 3.4 Statistical Power and Sample Size

Although simulation studies have provided useful insights into factors influencing model performance, there is no consensus on the minimum sample size requirements for LGM (Wang et al. 2021). Several data features are known to compromise the accuracy of class enumeration, class assignment, and parameter estimation, including small sample sizes (< 250) (Diallo et al. 2017), a limited number of time points (< 4) (Diallo et al. 2017), low class separation, high rates of missing data, and increased model complexity (Van Der Nest et al. 2020). A simulation study found that a minimum sample size of 200 was sufficient under conditions of complete data, four time points, and high class separation for a two‐class GMM. In contrast, a sample size of 800 was required when data featured 20% missingness and low class separation, though this could be reduced to 300 with the inclusion of up to 10 time points (Kim 2012). Furthermore, incorporating covariates strongly associated with class membership has been shown to improve class separation and reduce sample size requirements (Wang et al. 2021). Given earlier findings from the PEPP‐Montreal cohort (Percie du Sert et al. 2025), which identified 2‐ to 3‐class solutions with high class separation for 2‐year trajectories of functional and clinical outcomes in a sample of 689 individuals, and considering the planned inclusion of 4 to 10 time points and relevant covariates, a minimum sample size of 250–300 participants is expected to provide sufficient statistical power and reduce the risk of inaccurate class enumeration, misclassification, and biased parameter estimates.

## 4 Data Management and Open Science

Research assistants, all of whom hold at least a bachelor's degree in a mental health–related field, undergo comprehensive training provided by a research coordinator holding at least a master's degree. Prior to conducting assessments, they are required to achieve satisfactory inter‐rater reliability with expert consensus ratings. To maintain reliability over time, formal inter‐rater training sessions are held twice annually, and reliability is monitored using intra‐class correlation coefficients (ICCs). Continuous supervision is provided throughout the study, with weekly meetings to ensure strict adherence to protocol and resolve any ambiguities in data collection. To minimize bias, particularly expectation bias, and preserve the validity of the assessments, raters remain blind to diagnostic information and previous clinical ratings.

To ensure robust and transparent data management, the study utilizes the Castor Electronic Data Capture (EDC) platform, which facilitates secure data entry, documentation, and version control of the database. (Castor EDC 2019). A quality control procedure is implemented to monitor data integrity, with routine checks for missing values and validation against predefined ranges where applicable. Data quality is assessed in near real time, enabling the research team to promptly identify issues and, when necessary, request that a participant repeat a problematic measure, thereby ensuring data accuracy and completeness.

The PEPP10+ study is committed to open science practices by implementing data governance procedures aligned with the FAIR data principles (i.e., Findable, Accessible, Interoperable, and Reusable) (Wilkinson et al. 2016; Bacellar et al. 2023). In partnership with Maelstrom Research (Bergeron et al. 2018), the study adopts standardized approaches to documenting and disseminating metadata and data dictionaries to facilitate data discovery and harmonization. Researchers may request access to individual‐level de‐identified research data (excluding health administrative data, which remain the propriety of the Quebec Government) collected during the study. Access will be granted upon appropriate approvals, in accordance with the PEPP‐Montreal Early Psychosis Databank (Zavaglia et al. 2025) Data Access Policy.

## 5 Conclusion

The PEPP10+ study is uniquely positioned to test a foundational assumption of EIS, that early intervention during the critical period following the onset of psychosis can yield lasting effects. Specifically, the study examines whether the clinical and functional trajectories shaped during the initial 2 years of EIS are sustained over the long term, thereby evaluating the durability of early gains well beyond the period of specialized care. PEPP‐Montréal is a well‐established, high‐fidelity EIS, operating within a universal healthcare system and an epidemiologically defined catchment area. From the outset, it has integrated a robust clinical research program and implemented a comprehensive longitudinal assessment protocol, allowing for the detailed characterization of a large, representative, treated‐incidence sample of individuals with FEP. This 10‐year follow‐up assessment spans social, mental, cognitive, and physical health dimensions, offering a holistic perspective on long‐term outcomes. The 10‐year follow‐up assessment will be supplemented through linkage with health administrative databases, enabling continuous investigation of healthcare trajectories while helping to mitigate the impact of attrition. To our knowledge, this represents the most comprehensive investigation of long‐term outcomes in the context of EIS in North America. Precision psychiatry is an emerging approach that has so far offered limited prognostic guidance to patients with FEP, and few practical recommendations for clinicians on how to tailor EIS to individual profiles and needs. By adopting a data‐driven approach, the PEPP10+ study aims to capture the diversity of individual experiences and model the heterogeneity of long‐term outcome trajectories. This will help identify clinically meaningful subgroups of patients and early predictors of long‐term outcomes, while providing insight into the mechanisms and timing of changes within trajectories. In doing so, this study lays essential groundwork for the optimization of EIS and future development of personalized interventions.

## Funding

This study is supported by the Canadian Institutes of Health Research (180502). Salary awards include Canadian Institutes of Health Research (J.G., J.S., M.L.), the Canada Research Chairs program (K.K.A., S.I.), Fonds de Recherche du Québec en Santé (O.P.D.S., V.M., J.S., M.L.), and James McGill Professorship (M.L.). The funding source had no role in the writing and publication of the manuscript.

## Conflicts of Interest

R.J. reports receipts of grants, speaker's honoraria, and consultant's honoraria from AstraZeneca, Bristol Myers Squibb, HLS Therapeutics, Janssen, Lundbeck Canada, Myelin and Associates, Otsuka Canada, Purdue, Pfizer, Shire, and Sunovion. A.M. reports receipts of grants, speaker's honoraria, and consultant's honoraria from Bristol Myers Squibb Canada and Otsuka Lundbeck Alliance. M.L. reports receipt of grants from Otsuka Lundbeck Alliance, diaMentis, and Roche, personal fees from Otsuka Canada, Lundbeck Canada, and Boehringer Ingelheim, as well as grants and personal fees from Janssen. The other authors declare no conflicts of interest.

## Supporting information


**Table S1:** Selection criteria.

## Data Availability

The data that support the findings of this study are available on request from the corresponding author. The data are not publicly available due to privacy or ethical restrictions.
