# Supplementary material for: Long‐Term Trajectories of Multidimensional Outcomes in Psychosis Following Early Intervention During the Critical Period: The PEPP‐Montreal 10+ Study Protocol
Source: Early Interv Psychiatry. 2026 Sep 23;20(9):e70258. doi: 10.1111/eip.70258 (PMC13600794; doi:10.1111/eip.70258)
Supplement: Supplementary file 1 [file EIP-20-0-s001.docx]

**Supplementary Table 1. Selection criteria**

|  | **PEPP-Montreal Cohort** | **Non-EIS Controls** | **Mental Health Controls** | **Physical Health Controls** |
| --- | --- | --- | --- | --- |
| **Selection method** | Admission | Administrative data | Administrative data | Administrative data |
| **Selection period** | 2003 - 2018 | 2003 - 2018 | 2003 - 2018 | 2003 - 2018 |
| **Extraction period** | Date of admission to date of extraction (2023) | 10 years following date of index diagnosis | 10 years following date of index diagnosis | 10 years following date of index diagnosis |
| **Sample size** | 689 | 1670 | 690 | 690 |
|  | ≃ 50 admission / year | all cases identified | 46 individuals / year (x15) | 46 individuals / year (x15) |
| **Age** | 14 - 35 | 14 - 35 | 14 - 35 | 14 - 35 |
| **Residence** | Montreal West-Island | Montreal West-Island | Montreal West-Island | Montreal West-Island |
| **Index Diagnosis** | Schizophrenia and other psychotic disorders Mood disorders with psychotic features | SSD | Random psychiatric diagnosis, no lifetime psychotic disorder | Random medical diagnosis, no lifetime psychiatric disorder |
|  | DSM-IV (SCID) | ICD-9: 295[0 - 9], 297[0 - 3, 8, 9],  298[0 - 4, 8, 9]; ICD-10: F20 - F29 | ICD-9: 290 - 319 ICD-10: F00 - F99 | ICD-9: 001 - E999 ICD-10: A00 - U85 |
|  | Fist-episode: < 30-day antipsychotic use | First lifetime presentation of index diagnosis during selection period, such as associated to:  - 1 hospitalization (MED-ECHO), or  - 2 ER visits within same year (BDCU), or  - 2 medical visits within same year (RAMQ-MOD) | First lifetime presentation of index diagnosis during selection period, such as associated to:  - 1 hospitalization (MED-ECHO), or  - 2 ER visits within same year (BDCU), or  - 2 medical visits within same year (RAMQ-MOD) | First lifetime presentation of index diagnosis during selection period, such as associated to:  - 1 hospitalization (MED-ECHO), or  - 2 ER visits within same year (BDCU), or  - 2 medical visits within same year (RAMQ-MOD) |
| **Exclusion criteria** | Organic psychosis | PEPP-Montreal patients | SSD (ICD-9 295[0 - 9], 297[0 - 3, 8, 9], 298[0 - 4, 8, 9], 301.22; ICD-10: F20 - F29); | Mental and behavioural disorders (ICD-9: 290-319;  ICD-10: F00-F99) |
|  | Substance induced psychosis |  | Substance induced psychosis  (ICD-9: 291[2, 3, 5, 9], 292[1, 82, 9]; ICD-10: F10 - F19[5, 7]) | Individuals from other cohorts |
|  | CNS disorder |  | Organic psychosis  (ICD-9 : 293[81, 82, 89]; ICD-10 F06[0 - 2]) |  |
|  | Unable to consent |  | Individuals from other cohorts |  |
|  | IQ < 70 |  |  |  |
|  | Unable to speak either English or French |  |  |  |
